# Case Report: Diagnosis of Petrous Apex IgG4-Related Disease by Middle Cranial Fossa Craniotomy and Temporal Bone Biopsy

**DOI:** 10.3389/fneur.2022.874451

**Published:** 2022-06-09

**Authors:** Louis Hofmeyr, Gerrida Herbst, Elias Pretorius, Brian Sarembock, Kathryn Taylor, David Roytowski

**Affiliations:** ^1^Netcare Christiaan Barnard Memorial Hospital, Cape Town, South Africa; ^2^Division of Otorhinolaryngology, Tygerberg Academic Hospital, University of Stellenbosch, Cape Town, South Africa; ^3^Netcare Blaauwberg Hospital, Cape Town, South Africa; ^4^Morton and Partners Radiologists, Cape Town, South Africa; ^5^Pathcare, Cape Town, South Africa; ^6^Division of Neurosurgery, Tygerberg Academic Hospital, University of Stellenbosch, Cape Town, South Africa

**Keywords:** IgG4-related disease, temporal bone, middle cranial fossa, diplopia, case report, Gradenigo syndrome

## Abstract

**Introduction:**

Primary IgG4-related disease (IgG4-RD) of the temporal bone is a rare condition. Unlike typical petrous apicitis or Gradenigo syndrome, our patient presented exclusively with unilateral cranial nerve VI palsy and symptoms of diplopia. Skull base imaging demonstrated a destructive bony lesion in the petrous apex. Imaging and systemic investigations were insufficient to support a diagnosis. The diagnosis was achieved histologically after acquiring the specimen by middle cranial fossa craniotomy and temporal bone biopsy. This case report is thought to be the first published description of a diagnosis of IgG4-RD proven with the middle cranial fossa approach.

**Case Report:**

We describe a 29-year-old female with primary IgG4-RD of the petrous apex of the temporal bone. This patient presented with a few-month history of left-sided headache and recent-onset diplopia due to paralysis of cranial nerve VI. Imaging demonstrated a petrous apex lesion, and comprehensive systemic investigations could not reach a diagnosis. A middle cranial fossa craniotomy and a biopsy of the temporal bone lesion were undertaken to establish the diagnosis. Histological confirmation of IgG4-RD was proven. Following treatment with corticosteroids, the patient experienced complete recovery and resolution of her symptoms.

**Conclusion:**

This study describes a case of primary IgG4-RD of the petrous apex of the temporal bone that presented with diplopia and was diagnosed by middle fossa craniotomy and temporal bone biopsy. To the best of our knowledge, this is the first case description where primary diagnosis was made based on middle cranial fossa craniotomy and temporal bone biopsy.

## Introduction

IgG4-related disease (IgG4-RD) is a chronic immune-mediated fibro-inflammatory disorder (1). Perhaps, it is more common than anticipated, Although it was initially described in the context of autoimmune pancreatitis, it has now been described in nearly all parts of the body (2–4). It appears that IgG infiltration causes organ enlargement. Kuttner's tumor, Mikulicz's syndrome, and Riedel's thyroiditis are diseases that fall in the IgG4-RD spectrum (5–7). In the head and neck region, IgG4-RD of the thyroid, lymph nodes, nasal cavity, paranasal sinuses, lacrimal glands, orbits, and salivary glands have all been described (8–14).

Skull base involvement of IgG4-RD is less commonly noted, with most accounts being an extension from paranasal disease. IgG4-RD should be a consideration when a lesion involves the temporal bone in patients presenting with otalgia and diplopia (15–17).

Recently, a rare case of a patient with IgG4-RD of the temporal bone who presented with otalgia and diplopia due to cranial nerve VI palsy was described (18). Unsurprisingly, the presentation was considered Gradenigo syndrome, where otitis media and mastoiditis lead to temporal bone apicitis with otalgia and cranial nerve VI palsy (19). In this case, the diagnosis was made after a biopsy of the middle ear, mastoid, and sinus cavity (18). In another case, a patient with visual loss, facial nerve paralysis, and diplopia was diagnosed with IgG4-RD after obtaining histology via a mastoidectomy (20).

In this report, we present a young patient who had primarily diplopia as a symptom. This case is unique, as to the best of our knowledge, it is the first case of IgG4-RD diagnosed from a biopsy acquired with the middle cranial fossa approach. The serum IgG4 level in this patient was normal.

## Case Report

An otherwise healthy 29-year-old female presented to the emergency room with diplopia from isolated cranial nerve VI palsy on the left and a history of left-sided headaches of several-month duration. Associated symptoms included pressure in the left ear and a “blocked” sensation. The patient did not complain of any vestibular symptoms. Otoscopy revealed normal tympanic membranes with no signs of middle ear pathology. Diagnostic audiometry revealed normal hearing and middle ear function. Normal blood investigation findings included fasting glucose, corrected calcium, magnesium, liver functions, thyroid functions, and a complete blood and platelet count. Serological testing, including human immunodeficiency virus (HIV) testing, treponema pallidum antibodies, anti-nuclear antibody (ANA) titers, and antineutrophil cytoplasmic antibody (ANCA) titres, was negative. Erythrocyte sedimentation rate (ESR) was 29, and C-reactive protein (CRP) was 14, mildly raised in both instances.

**Figure 1:**
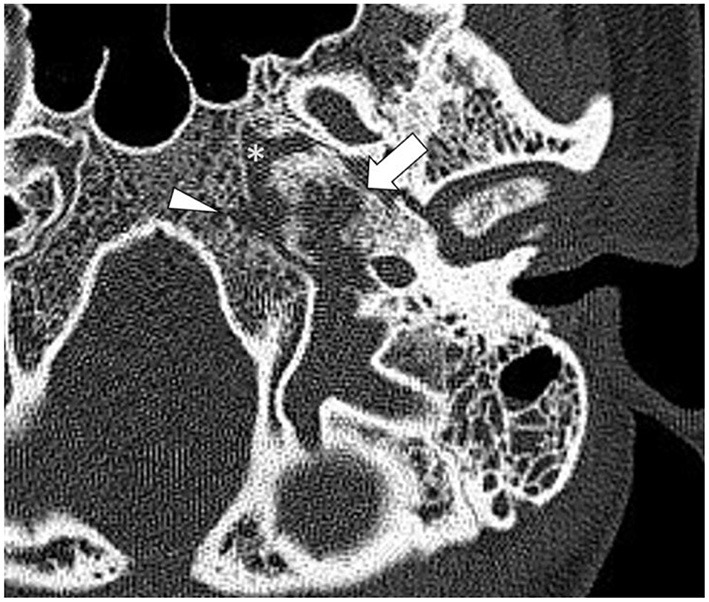
Axial high-resolution computed tomography (HRCT) of the skull base shows a destructive process in the petrous apex (arrow) with regional sclerosis involving the petroclival fissure (asterisk) and clivus (arrowhead).

**Figure 2:**
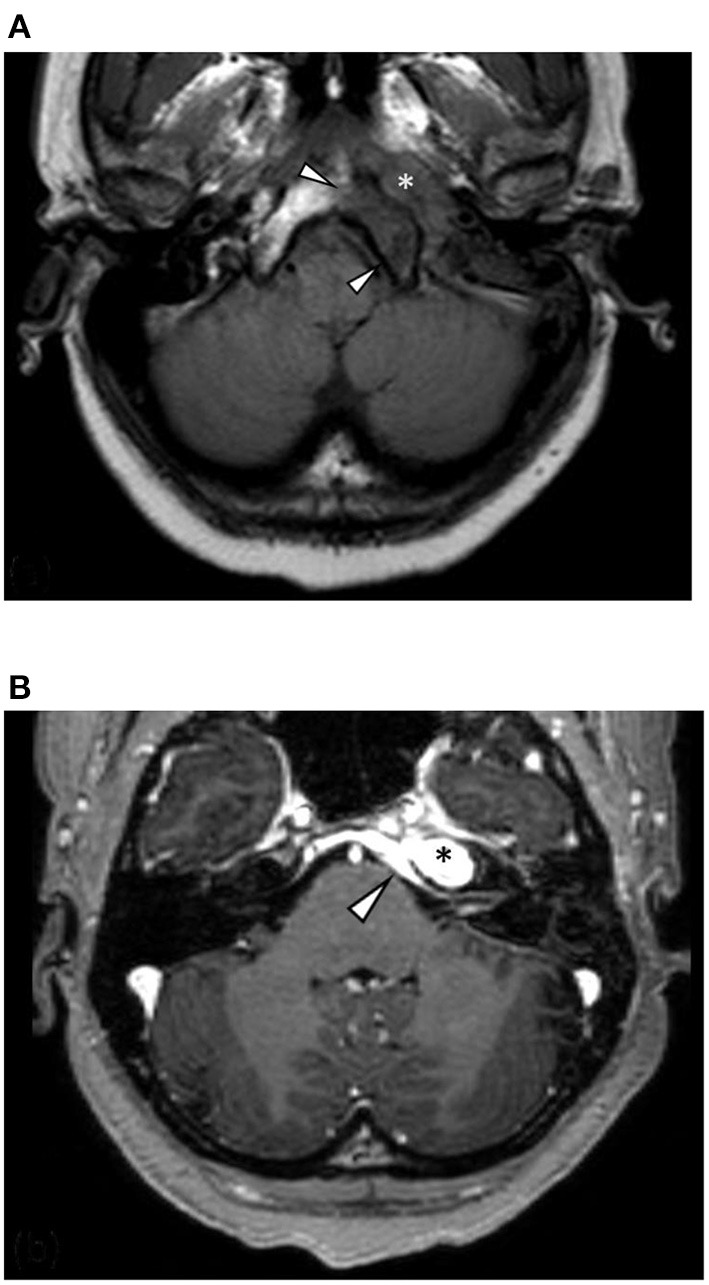
**(A)** Axial T1-weighted MRI shows a hypointense mass centered on the left petrous apex (asterisk) and regional marrow hypointensity due to infiltration and marrow replacement (arrowheads). **(B)** Axial T1 following contrast administration shows avid homogenous enhancement of the lesion (asterisk) and intracranial disease extension with pachymeningeal enhancement and thickening of the retroclival dura (arrowhead).

High-resolution computed tomography (HRCT) of the skull base demonstrated an aggressive erosive disease process centered on the left petrous apex involving the petroclival fissure and inferolateral clivus (Figure 1). Subsequent magnetic resonance imaging (MRI) revealed a trans-spatial marrow-replacing tumor-like soft tissue lesion of low T1/T2 signal (Figure 2A) and mild apparent diffusion coefficient (ADC) reduction. The lesion was enhanced avidly following gadolinium contrast administration (Figure 2B). Focal pachymeningeal thickening of the retroclival dura and enhancement of the cisternal segment of the left abducens nerve as it enters Dorello's canal were present.

The initial diagnosis favored Gradenigo syndrome, otomastoiditis, lateral rectus palsy, and deep facial pain, with a differential diagnosis of cholesteatoma, cholesterol granuloma, tuberculosis, mucocele formation, and petrous apex air cell effusion. The absence of signs of a systemic disease, clinically and later supported by pathology results, ruled out an infective process. Malignancies considered included chondrosarcoma, chordoma, plasmacytoma, multiple myeloma, paraganglioma, and meningioma. A tissue biopsy was required to make a definitive diagnosis.

Surgical access to the petrous apex includes the infracochlear, infralabyrinthine, middle fossa, and transsfenoid approaches. After studying the anatomy on the HRCT scans, the middle fossa approach was chosen as the most appropriate option.

**Figure 3:**
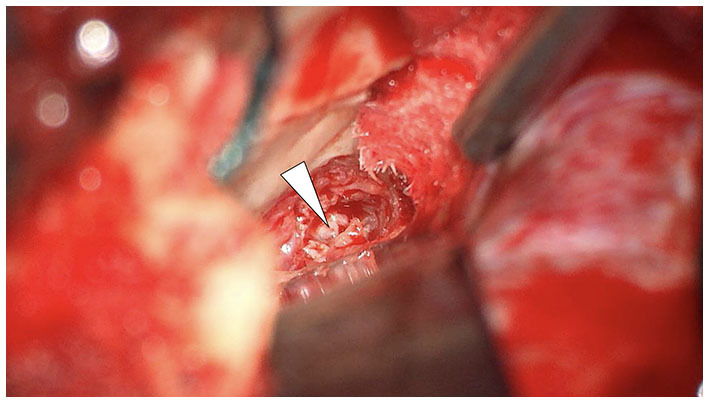
Middle cranial fossa craniotomy demonstrating the lesion in the petrous apex (arrowhead).

Given the epicenter of the lesion, middle fossa craniotomy provided an elegant and effective means of approaching the lesion to obtain tissue for histological analysis. Nevertheless, the middle cranial fossa approach to the temporal bone can be considered invasive and is not without risks. However, by employing the transtemporal supralabyrinthine modification of the middle cranial fossa approach, described by Fisch, minimal retraction is placed on the temporal lobe (21, 22). In the author's experience of more than 150 cases, the risk of developing epilepsy with this modification is minimal. Following the craniotomy, the petrous apex was identified, image guidance utilized to confirm and diamond burr drilling of the distorted petrous bone performed to access the lesion. The macroscopic appearance of the tissue was pink, firm, and without a clear capsule or vascularity (Figure 3). Numerous tissue biopsies were taken. The patient recovered well after the surgery without complications.

**Figure 4:**
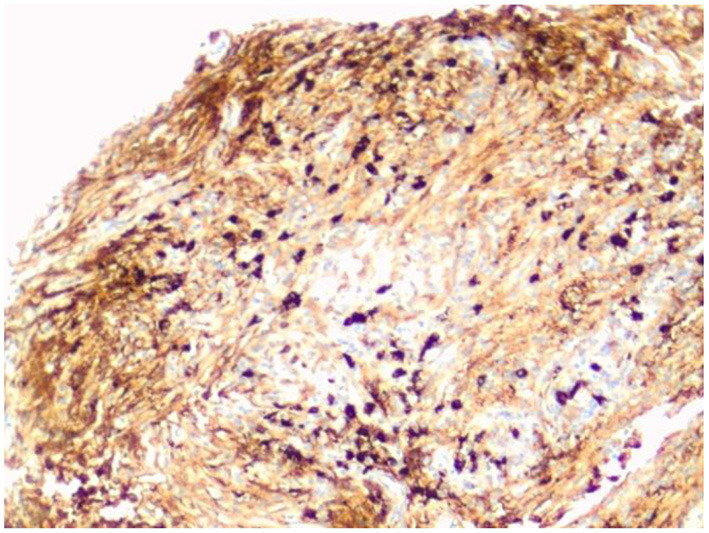
IgG4 immunohistochemical stain highlights the IgG4-positive plasma cells. There are more than 50 IgG4-positive cells in this field (magnification × 200).

The microscopic examination of the biopsy fragments showed a dense lymphoplasmacytic inflammatory infiltrate with a fibrous background. Strands and vague fascicles of collagen fibers were also seen, as well as regions of focal storiform-type fibrosis. There was no evidence of obliterative phlebitis or granulomata. Fungal, bacterial, and mycobacterial stains were negative. A lymphoproliferative disorder was excluded. Immunohistochemical stains for IgG4 showed greater than 50 IgG4-positive plasma cells in some high-power fields (Figure 4). IgG4/IgG ratio was greater than 20%. These features were highly suggestive of IgG4-related disease.

Once the diagnosis was made, the patient was commenced on oral prednisone at 40mg daily to taper over a 6-month interval.

One month post-surgery, the patient complained of hoarseness. She was diagnosed with left vocal cord paralysis, with the affected vocal cord in the paramedian position. MRI revealed an extension of the original disease process, and the MRI of the neck was normal. In the light of the MRI findings, the paralysis of the recurrent laryngeal nerve was thought to be due to IgG4-related peripheral axonal neuropathy.

Three months after the post-procedure, the patient continued on prednisone. The left cranial nerve VI palsy and vocal cord paralysis have resolved entirely. The patient returned to work with an occasional complaint of blurred vision. Concerns exist that upon discontinuation of prednisone, she will experience symptom recurrence.

## Discussion

IgG4-RD is a recently described systemic fibro-inflammatory disease, although it was first diagnosed in salivary glands (Mikulicz's disease) more than 100 years ago (7). The etiology remains unclear, although the most likely mechanism is thought to be antigen-driven inflammation or infection (1, 5).

The head and neck region is the second most common site of IgG4-RD presentation (1). This case report is, to our knowledge, the first from the African continent. IgG4-RD involvement of the ear is thought to be rare. The sexual preponderance is roughly equal (2, 3, 5). It occurs mainly in patients in the 6^th^ decade (1). Our case was somewhat unusual, as the patient was 29 years old.

Diagnosis is usually made by correlating clinical presentation, serological studies, imaging findings, and histopathological results (2). Histological confirmation of the diagnosis is the gold standard (4).

IgG4-RD may mimic many conditions, including infections, malignancies, and autoimmune diseases (5). Clinicians should cultivate a healthy index of suspicion in patients with atypical presentation.

Although the head and neck are often involved in systemic IgG4-related disease with frequent lacrimal and major salivary glands, primary skull base involvement is highly uncommon. The typical imaging features of a skull base disease are nonspecific and often mimic inflammatory and neoplastic entities. Focal lesions are frequently misinterpreted as nasopharyngeal carcinoma, meningioma, or primary lymphoma and are usually included in the imaging differential diagnosis (22–24).

The typical imaging characteristics of IgG4-RD describe a focal mass-like and infiltrative lesion with iso- to hypointense signal intensity on T2 and correlative apparent diffusion coefficient (ADC) reduction. Imaging features may vary in intensity depending on the degree of fibrosis and lymphoplasmacytic cellular composition. Lesions are typically enhanced homogeneously following contrast administration. HRCT of the skull base is a helpful adjunct to MRI and superior in assessing osseous destruction and erosion (24–27).

IgG4-RD is an immune-mediated inflammatory disorder in multiple organs characterized by abundant infiltration of IgG4-positive plasmacytes and fibrosis in the involved organs. The precise pathogenic mechanism remains unclear. Aberrant innate and adaptive immunity is considered the primary pathogenesis of IgG4-RD.

Recent studies have shown that abnormal adaptive immune responses mediated by T-helper type 2 cells, regulatory T lymphocytes, CD4 -positive cytotoxic T lymphocytes, T follicular helper cells, and T follicular regulatory cells are involved in IgG4-RD (5, 28). In addition to adaptive immune responses, innate immune responses play pathogenic roles in IgG4-RD. Macrophages, mast cells, basophils, and complement and plasmacytoid dendritic cells are activated to produce various cytokines in IgG4-RD. There is increasing evidence that B cells play significant roles in the pathogenesis of IgG4-RD. The fact that B cell depletion has been shown to correlate with rapid improvement of tissue fibrosis in affected organs of patients with IgG4-RD raises the hypothesis that B lymphocytes may be involved in fibrogenesis.

Currently, the standard of care for IgG4-RD worldwide remains to be corticosteroids (prednisone). Treatment usually starts with 40 mg of prednisone daily for 1 month. This dose is then tapered to and maintained at 5 to 10 mg per day. Should the steroids fail, rituximab should be considered as a second-line treatment (29).

Making the diagnosis of IgG4-RD requires tissue sampling; if the petrous apex is the site of infiltration, middle cranial fossa craniotomy can be performed. The middle cranial fossa craniotomy approach may be considered invasive. Temporal lobe epilepsy is a known potential complication. It is based on the author's experience in performing over 150 cases of middle cranial fossa approaches that this is an infrequent occurrence when the modified transtemporal supralabyrinthine approach is employed, as described by Fisch. The technique involves drilling the temporal bone to access the petrous apex rather than retracting the temporal lobe. The technique has been described as “one-third temporal lobe retraction and two-third temporal bone drilling”. The important takeaway message is that middle cranial fossa craniotomy with transtemporal supralabyrinthine modification, as described by Fisch, is a viable, safe, and practical approach to obtain access to the petrous apex (21, 22).

## Conclusion

In summary, we describe, to our knowledge, the first case of primary temporal bone IgG4-RD causing cranial nerve VI palsy that has been proven histologically by a middle cranial fosssa craniotomy and temporal bone biopsy. This demonstrates that the middle cranial fossa approach, which is considered invasive, is a practical and viable option to diagnose petrous apex lesions.

## Data Availability Statement

The raw data supporting the conclusions of this article will be made available by the authors, without undue reservation.

## Ethics Statement

Written informed consent was obtained from the individual(s) for the publication of any potentially identifiable images or data included in this article.

## Author Contributions

LH: otorhinolaryngologist involved in the middle cranial fossa biopsy, coordinator of contributions, submission, and contact for the manuscript. DR: neurosurgeon involved in the middle cranial fossa craniotomy, preparation, and grammar check of the manuscript. GH: primary treating otorhinolaryngologist. BS: treating rheumatologist. EP: reporting radiologist. KT: reporting histopathologist. All authors contributed to the article and approved the submitted version.

## Conflict of Interest

The authors declare that the research was conducted in the absence of any commercial or financial relationships that could be construed as a potential conflict of interest.

## Publisher's Note

All claims expressed in this article are solely those of the authors and do not necessarily represent those of their affiliated organizations, or those of the publisher, the editors and the reviewers. Any product that may be evaluated in this article, or claim that may be made by its manufacturer, is not guaranteed or endorsed by the publisher.
